# Cardiac Hydatid Cyst: A Rare but Potentially Life-Threatening Presentation of Hydatid Disease

**DOI:** 10.14797/mdcvj.1333

**Published:** 2024-03-14

**Authors:** Kanhai Lalani, M. Sudhakar Rao, R. Padmakumar, Pankti Parikh, M. V. Ashwini, Ujwal teja Dhulipalla

**Affiliations:** 1 Kasturba Medical College, Manipal, Manipal Academy of Higher Education, Manipal, Karnataka, India

**Keywords:** hydatid disease, cardiac hydatid cyst, Echinococcus species, albendazole, echinococcosis, hydatid cyst

## Abstract

Cardiac echinococcosis is a rare and severe manifestation of hydatid disease. It is caused by parasitic infestation by the *Echinococcus* species and can lead to life-threatening complications. Diagnosis is difficult due to nonspecific symptoms, but echocardiography is a highly sensitive diagnostic method. Albendazole treatment is effective in managing these cysts and can be an alternative to surgery. A patient with multiple cardiac hydatid cysts was successfully treated with albendazole, highlighting the importance of prompt diagnosis and treatment to prevent life-threatening complications.

## Description

Hydatid disease is caused by the *Echinococcus* parasite and is commonly found in sheepherding regions such as India. Humans can become infected by inadvertently ingesting tapeworm eggs from contaminated food and water.^1,2^ The disease mainly affects the liver and lungs, with cardiac involvement observed in less than 2% of patients. Hydatid cysts are predominantly found in the left ventricle (55-60%), followed by the right ventricle (10-15%), pericardium (7%), pulmonary artery (6-7%), left atrium (5%), right atrium (3-4%), and interventricular septum (4%).^1,2^ Most patients remain asymptomatic, but 10% may present with clinical symptoms depending on the cyst’s location, size, and expansion. Complications may include systemic or pulmonary embolism, pericarditis, valvular obstruction or regurgitation, and anaphylaxis.^3,4^ Cardiac hydatid cysts are challenging to diagnose due to nonspecific symptoms and electrocardiographic (ECG) findings. Echocardiography is a reliable diagnostic modality for cardiac hydatid cysts.^1,3,4^ A combined surgical resection and concurrent albendazole treatment yield excellent outcomes.^1,2^

**Figure 1:**
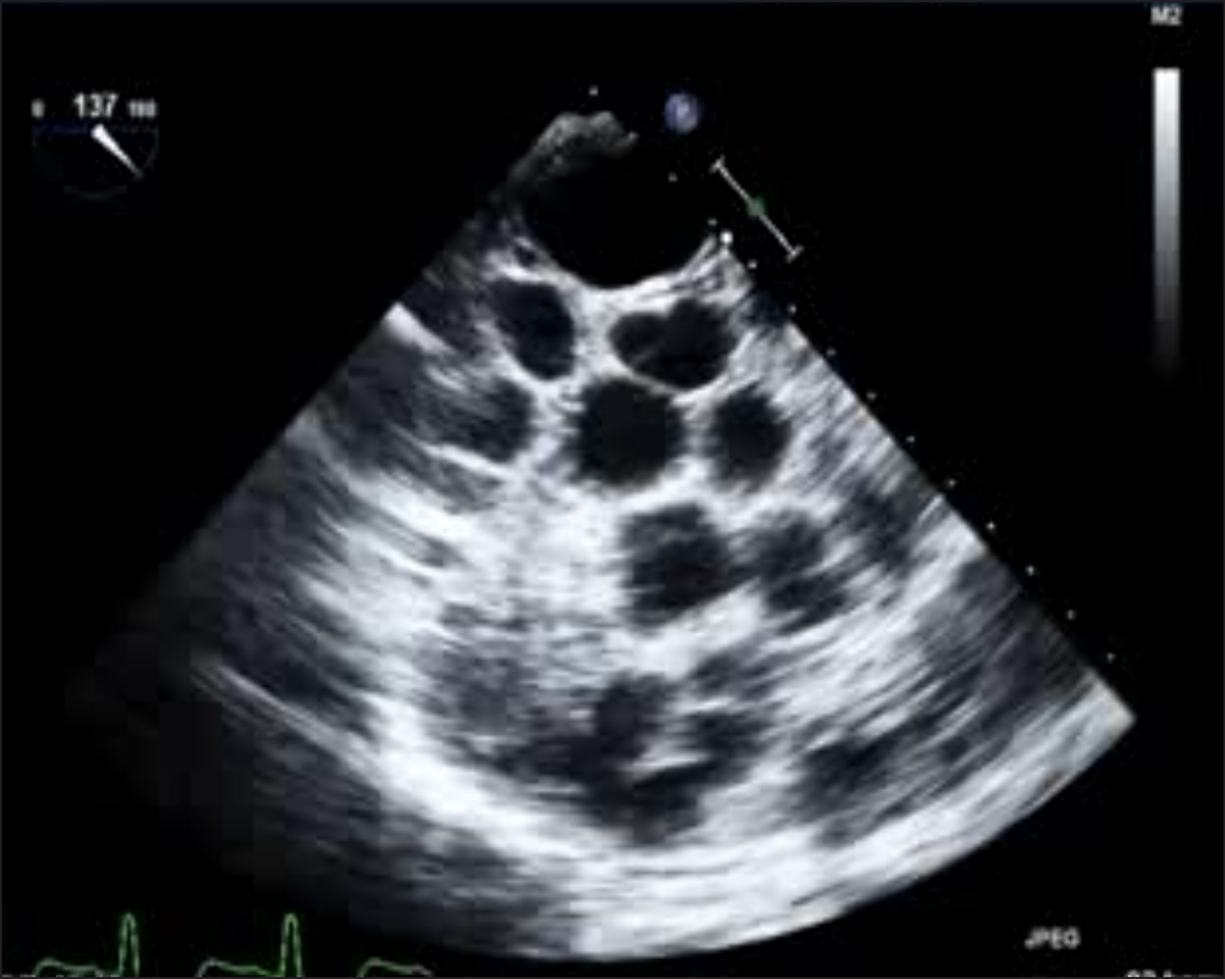
A transesophageal echocardiogram reveals the presence of numerous hydatid cysts entirely filling the left atrium and left ventricle cavity.

**Figure 2:**
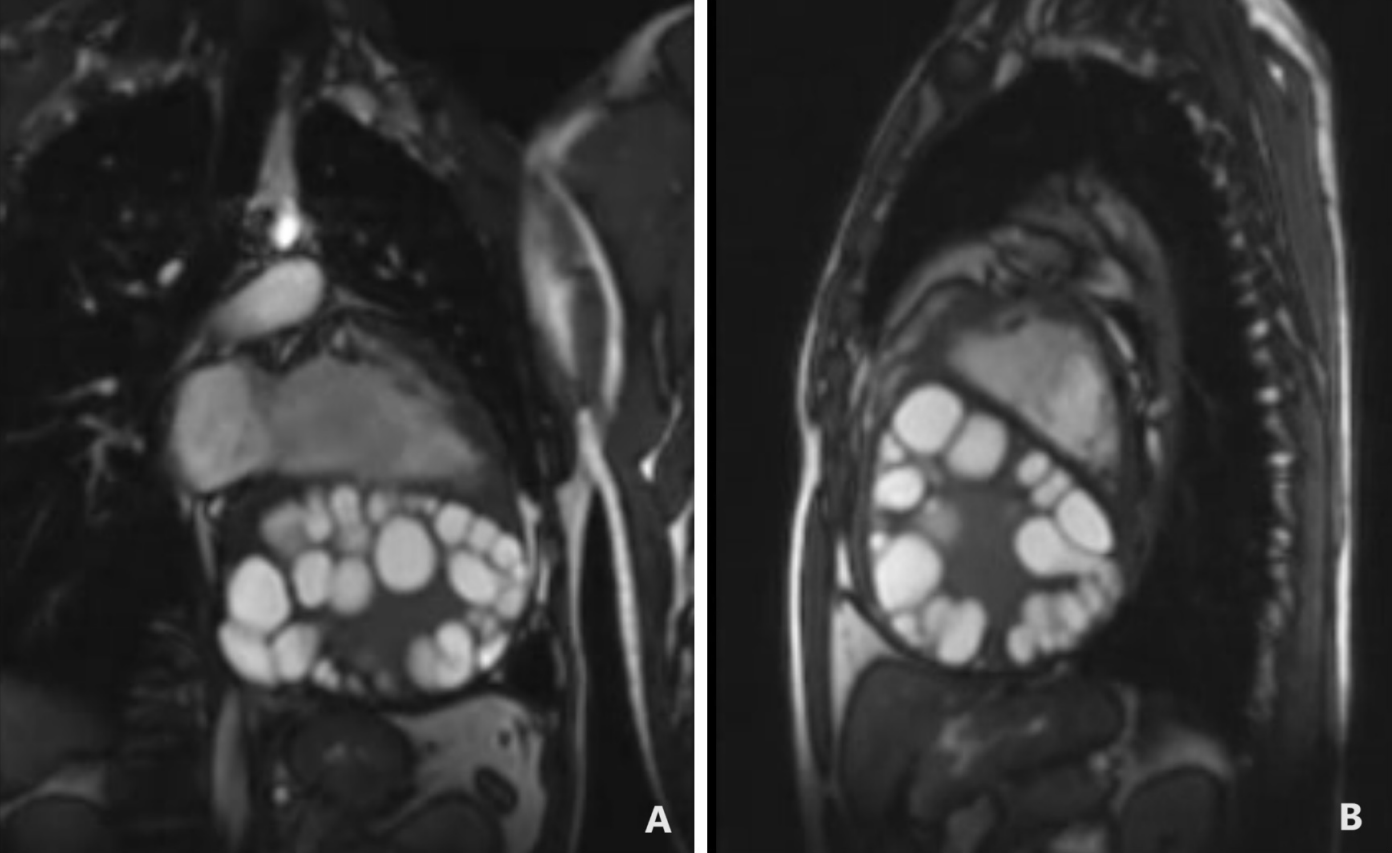
Cardiac magnetic resonance imaging through static images of a cine loop. **(A)** The sagittal section features a four-chamber view, while **(B)** displays a short-axis view. Both images depict multiple hypointense ring-shaped thick-walled hydatid cysts occupying the entire cavity of the left atrium and left ventricle.

A man in his sixties presented with chest discomfort for several weeks. His ECG showed probable ischemic T inversions in inferolateral leads, and coronary angiography revealed normal epicardial coronaries with slow flow. Echocardiography revealed multiple echolucent cysts, with septa occupying the entire left atrium (LA) and left ventricle (LV), along with a normal ejection fraction. Transthoracic echocardiography images were not included due to an unclear window and poor image quality resulting from the patient’s obesity. Transesophageal echocardiography showed a loculated mass in the LV myocardium measuring 34 × 35 mm with an extension of about 52 × 42 mm, compressing the LA (Figure 1, Video 1). Positive serology results for echinococcosis were obtained. Magnetic resonance imaging confirmed a large cystic mass with septa in the left atrium and LV, suggestive of a cardiac hydatid cyst (Figure 2, Video 2). The patient opted for albendazole (400 mg twice a day) for 1 year instead of surgery. After a year, cysts disappeared completely, demonstrating albendazole as a viable alternative for surgery.

**Video 1:** A transesophageal echocardiogram reveals the presence of numerous hydatid cysts entirely filling the left atrium and left ventricle cavity; see also at https://youtu.be/QBTMjZTEpc8.

**Video 2:** Cardiac magnetic resonance imaging, cine loop. The sagittal section and short-axis view showed multiple hypointense ring-shaped thick-walled hydatid cysts occupying the entire cavity of the left atrium and left ventricle; see also at https://youtu.be/zaIBK_WH57M. LA: left atrium; LV: left ventricle
